# Use of an electronic wellness instrument in the integrated health and social care of older adults: a group concept mapping study

**DOI:** 10.1186/s12913-024-11320-5

**Published:** 2024-07-30

**Authors:** Melissa Northwood, Margaret Saari, George Heckman, Ted Alexander, Bill Eastway, Patricia Gerantonis, Deanne Gillies, Susie Gregg, Jane McKinnon Wilson, Adam Morrison, Heebah Sultan, Luke Turcotte

**Affiliations:** 1 School of Nursing, Faculty of Health Sciences, McMaster University, Health Sciences Centre 3N25a, 1280 Main Street West, Hamilton, ON L8S 4K1 Canada; 2 SE Research Centre, SE Health, Markham, ON Canada; 3 School of Public Health Sciences, University of Waterloo, Waterloo, ON Canada; 4 eHealth Centre of Excellence, Kitchener, ON Canada; 5 Patient Partner, Guelph, ON Canada; 6 K-W Seniors Day Program, Kitchener, ON Canada; 7 Canadian Mental Health Association Waterloo Wellington, Guelph, ON Canada; 8 Provincial Geriatrics Leadership Ontario, Toronto, ON Canada; 9 Ontario Health, Toronto, ON Canada; 10 Health Sciences, Brock University, St. Catharines, ON Canada

**Keywords:** Aged, Care partners, Privacy, Multiple chronic conditions, Medical informatics, Confidentiality, interRAI, Self-report

## Abstract

**Background:**

Health system fragmentation directly contributes to poor health and social outcomes for older adults with multiple chronic conditions and their care partners. Older adults often require support from primary care, multiple specialists, home care, community support services, and other health-care sectors and communication between these providers is unstructured and not standardized. Integrated and interprofessional team-based models of care are a recommended strategy to improve health service delivery to older adults with complex needs. Standardized assessment instruments deployed on digital platforms are considered a necessary component of integrated care. The aim of this study was to develop strategies to leverage an electronic wellness instrument, interRAI Check Up Self Report, to support integrated health and social care for older adults and their care partners in a community in Southern Ontario, Canada.

**Methods:**

Group concept mapping, a participatory mixed-methods approach, was conducted. Participants included older adults, care partners, and representatives from: home care, community support services, specialized geriatric services, primary care, and health informatics. In a series of virtual meetings, participants generated ideas to implement the interRAI Check Up and rated the relative importance of these ideas. Hierarchical cluster analysis was used to map the ideas into clusters of similar statements. Participants reviewed the map to co-create an action plan.

**Results:**

Forty-one participants contributed to a cluster map of ten action areas (e.g., engagement of older adults and care partners, instrument’s ease of use, accessibility of the assessment process, person-centred process, training and education for providers, provider coordination, health information integration, health system decision support and quality improvement, and privacy and confidentiality). The health system decision support cluster was rated as the lowest relative importance and the health information integration was cluster rated as the highest relative importance.

**Conclusions:**

Many person-, provider-, and system-level factors need to be considered when implementing and using an electronic wellness instrument across health- and social-care providers. These factors are highly relevant to the integration of other standardized instruments into interprofessional team care to ensure a compassionate care approach as technology is introduced.

## Background

Integrated and interprofessional team-based care is recommended internationally as a strategy to strengthen health-care systems [1–7]. Implementing team-based models of care has received renewed attention after long-standing health system issues and health inequities were intensified and emphasized because of the COVID-19 pandemic [1, 4, 7]. Integrated care with a community-oriented focus, where older adults and health- and social-care providers partner to shape health-care delivery, can facilitate comprehensive and holistic care [4, 8]. Calls for a system re-design where care is delivered in a more compassionate way are born out of the experiences of many older adults (≥ 65 years) and their care partners, particularly those living with multiple chronic conditions. These individuals, who often require supports spanning multiple providers and health and social care sectors, often have their needs unmet because providers work in fragmented and uncoordinated silos [9, 10]. Communication between these providers is unstructured and unstandardized, and thus sub-optimal [5, 11]. Poor information sharing is further complicated by time-consuming over-assessment of some issues, and incomplete assessment of others, leaving less time available to understand the person behind the chronic conditions [12]. As a result, older adults continue to be exposed to preventable health crises, worsening quality of life, loss of independence, and care partner distress [9, 11].

An essential feature of integrated care includes the effective use of standardized assessment instruments [8, 11, 13, 14]. Standardized assessment instruments provide a common clinical language interpretable by all health- and social-care providers. When supported by interoperable digital tools and shared electronic health records, standardized assessments allow older adults, care partners, and providers to exchange information and create and share a common care plan [8–11, 14–17]. As integrated technologies are employed across health care systems, compassionate care—care that raises awareness of and responds to suffering—should be enhanced rather than impeded [18]. A standardized assessment, combined with an in-depth exploration of an individual’s narrative, are both essential for compassionate care [19]. Self-report tools provide an opportunity for an older person to describe functional needs, and their impact, and express concerns often ignored in health-care interactions, including mood, loneliness, financial hardship, food insecurity, and stressful life circumstances [5, 20, 21]. Efficiencies gained by using self-report tools to identify client concerns prior to health care interactions can be leveraged by the care team to better focus on the individual’s personal needs and goals, and thus develop a care plan most likely to alleviate suffering and support the older person and care partners throughout their health journey [5, 10, 19, 20].

The interRAI Check Up (hereafter referred to as the “Check Up”) is a 90-item instrument that supports assessment of a broader range of health and social care needs. interRAI is a not-for-profit international network of clinicians and researchers which develops and maintains an integrated family of instruments to assess vulnerable persons across settings, such as home care and long-term care [11]. The Check Up is a self-report instrument, deployed on a software platform, targeting community-dwelling older adults, and can be completed by non-clinicians or older adults themselves [22, 23]. It supports care planning and identifies areas of need (outputs) related to cognition, mood, loneliness, pain, instrumental and basic activities of daily living, falls, cardiopulmonary risk, care partner stress, financial trade-offs, health stability, and frailty [22]. The Check Up is based on the interRAI Home Care assessment, a clinician-driven instrument, which has demonstrated reliability, validity, and effectiveness in supporting care planning and fostering collaboration across the health-care system [11, 22, 24].

We have previously shown the feasibility of using a standardized self-report instrument for screening during the pandemic, the interRAI COVID-19 Vulnerability Screener (CVS), in community support services, primary care, and assisted living settings to identify vulnerable persons and refer them to the required social- and health-care services [25, 26]. The interRAI CVS was developed early in the COVID-19 pandemic based on items from the Check Up. Additionally, implementation of the Check Up in a geriatrician’s practice showed how the instrument helped flag, track, and prioritize all areas of need (social and medical) for immediate and future care planning [27].

In both of these studies, using standardized self-report instruments was determined to be feasible and have the potential to support better system integration, but suboptimal collaboration between community support services, specialized geriatric services, and primary-care providers persists, highlighting the need for more intentional planning around the use of these instruments [26, 27].

The purpose of this study was to develop strategies to leverage an electronic wellness instrument (the Check Up) to support more compassionate and integrated health and social care for older adults and their care partners. This study aimed to: (1) identify needs and factors related to using integrated technologies to share the outputs of an electronic wellness instrument across health- and social-care providers and (2) develop strategies to use this information to provide integrated, person-centred and compassionate health and social care to older adults with multiple chronic conditions and their care partners.

## Methods

### Study design

The study used group concept mapping (GCM), a participatory mixed methods approach, where participants share and organize ideas to identify issues and establish consensus on a framework for action [28, 29]. This design has been used extensively to consider the perspectives of community members in health and community-based research [20, 30, 31]. The GCM process involves five steps: (1) preparation, (2) idea generation, (3) sorting and rating, (4) generating maps and (5) interpreting and validating maps [28]. Groupwisdom™ software was employed to allow synchronous and asynchronous participation options and embedded analysis functions. The process occurred over a series of Zoom meetings (three with participants and four with the steering committee) from March to December 2022.

### Setting and participants

This study engaged a community in south-western Ontario, Canada of both urban and rural geography with a population of approximately 700,000 persons, 14.9% being ≥ 65 years [32]. A steering committee was formed with older adults, co-investigators, and organizational collaborators, such as the Alzheimer’s Society, eHealth Centre for Excellence, and home care, community supports, mental health, and primary care organizations. Steering committee members provided study oversight, participated in data analysis, contributed to generating an action plan, and shared this plan with their organizations. Older adults participated on the steering committee to ensure the relevance and applicability of the study outcomes to older adults.

A purposive sample of research participants were recruited through the professional networks of the steering committee members, using email and phone recruitment scripts. Persons were eligible to participate if they were an older adult (≥ 65 years), a care partner of an older adult, or staff or leader of an organization providing services to older adults, including home care, community support services, specialized geriatric services, primary care, and digital health/health informatics. Members of the steering committee (excluding co-investigators and the research assistant) were also invited to contribute to the group concept mapping activities. We anticipated a sample size of 28 to 34 participants, with a goal to include 10 older adult and/or care partner participants and three to four persons from each of the different sectors.

### Data collection and analysis

#### Step 1: preparation

A focus prompt was proposed by the research team and refined by the steering committee. In GCM, a focus prompt is used during idea generation to describe participants’ opinions regarding the study topic [28]. The prompt used during data collection was: “To support the use of the interRAI Check Up, a digital health tool, as part of a compassionate care approach with older adults, we should consider …”.

#### Step 2: idea generation

During the idea generation step, participants independently created responses to the focus prompt. The goal of this step was to generate diverse ideas, including the voices of all the different participants. Utilizing the software platform, participants entered their ideas and could view the ideas, in real-time, being shared by the other participants. This step was completed during a Zoom session (lasting two hours), facilitated by the research team, but participants could choose to participate independently outside of the meeting time. The idea generation activity was open for three weeks following the Zoom meeting to give participants ample time to contribute. Participants were also asked to complete a short, demographic survey to collect information about their sector or role.

A total of 215 ideas were generated by participants. Members of the research team (MN, MS, PG, SG, HS) conducted an iterative analysis to consolidate the generated ideas into a manageable set of less than 100 ideas, suggested as best practice in GCM [28]. This process involved editing statements for clarity and splitting statements if they contained more than one idea to ensure only one idea was represented per statement [28]. To aid in identifying duplicate ideas, a topic area was identified for each statement and statements were grouped by the topics to visualize potentially similar statements. The research team and members of the steering committee reviewed the consolidated statement set to ensure original ideas were captured, statements were clear and understandable, and each statement was unique. Changes to wording to enhance the clarity of some statements were suggested and incorporated. The final statement list contained 98 statements.

#### Step 3: sorting and rating

At a second data collection meeting over Zoom (lasting two hours), participants contributed to the sorting and rating activities using the online software platform. One participant elected to do this activity with a hardcopy version. During the sorting activity, participants were asked to independently group the statements into categories based on their similarities and label each group with a name that they felt reflected the statements comprising the grouping. Participants were provided with detailed instructions ahead of and during the meeting to sort the ideas into a minimum of five categories, and devise labels for each. Following the sorting activity, participants were asked to independently rate each statement according to its “importance” and their perceived “community capacity” to support use of the Check Up as part of a compassionate approach to care with older adults. Participants were directed to consider their community’s capacity to use the Check Up at any of the following levels: individual (e.g., knowledge, skill, trust), organization (e.g., human resources, leadership, policies), or health system (e.g., resource allocation, policies, collaborations). Each statement was rated by participants on a scale of one (not at all important/no capacity) to four (very important/full capacity). The sorting and rating activities were open for two weeks following the Zoom meeting for participants to complete.

#### Step 4: generating maps

We used the groupwisdom™ software to perform multidimensional scaling and hierarchical cluster analysis to depict the results of the sorting activity in map form [28, 33, 34]. First, a similarity matrix was created that reflects the number of participants who sorted each pair of statements together in the sorting activity [28]. Using multidimensional scaling analysis of the similarity matrix, a point map was created that locates each statement as a separate point on a two-dimensional map [28]. The quality of this analysis was verified using a stress index, which measures the discrepancy between distances of points on the map and their original value in the similarity matrix [28, 33]. A pooled analysis of GCM studies found the average stress value was 0.28 (SD = 0.04, range: 0.17-0.034, 95% CI [0.27, 0.29]) [33]. Each point represented an individual statement that was sorted by participants, with similar statements located closer to each other on the map and less similar statements located further apart [28].

Hierarchical cluster analysis was conducted to group individual statements into clusters, using Ward’s algorithm [28]. Agglomerative methods were used in the analysis, beginning with each statement, and merging them successively until they are in non-overlapping clusters [28]. This analytical approach produced multiple maps with a varying number of clusters. GCM methodology asserts that there is no definitive or “correct” number of clusters but rather the map is determined by the research team, in consultation with participants, to identify the number of clusters that yield conceptually meaningful and distinct domains [28, 33, 34].

A subgroup of the research team (MN, MS, PG) reviewed the possible cluster solutions, facilitated by the ‘cluster replay’ feature in the software, starting with a small number of clusters (five), and moving up to solutions with more clusters to review changing cluster compositions. Consideration was given to the logic of the clusters as a set and in the context of the other clusters [28]. Maps with nine, 10 and 11 clusters were shared with the steering committee. The decision to share these cluster solutions was based on discussion of what a reasonable number of cluster solutions to share during the meeting to facilitate consensus-building and most feasible to identify implementation strategies by cluster. By consensus, a 10-cluster solution was determined as the best fit to present to the participants for feedback and validation. The cluster names were reviewed and edited, considering the statements that were part of the cluster.

A go-zone graph was also generated, based on the importance rating data, to illustrate the prioritization of each idea according to importance. The purpose of including a go-zone map for this study was to understand the relative ratings of the statements within the clusters, identify initial priority issues to consider in developing strategies, and emphasize the perspectives of older adults. A go-zone graph is a bivariate plot that places each idea on a point, determined by its average rating of importance [28]. For this project, we created a go-zone graph that considered the importance as rated by older adults and the other participants. The go-zone graph is divided into four quadrants with the x-axis (older adults) and the y-axis (other participants), with ideas in the upper right quadrant representing the most important considerations for both groups.

#### Step 5: interpreting maps

At the final data collection meeting, the 10-cluster map was shared with participants. They were asked to provide their input on whether the statements were suitably grouped, if any statements should be sorted elsewhere, and if the cluster titles accurately reflected the statements. Three statements were relocated to adjacent clusters and some changes to cluster titles were suggested before consensus was reached on the map.

Participants also reviewed the go-zone graph and the set of statements that were rated as the highest relative importance. Participants were asked to identify which statements should be prioritized for action planning and those statements that reflected the vision of creating a compassionate community of digital health care. These recommendations were developed into an action plan by members of the research team, which included strategies to consider when sharing and using the outputs of the Check Up across health and social care sectors (MN, MS, PG, SG, AM, GH). The action plan was reviewed and discussed at the final steering committee meeting, and further refinements were made.

## Results

**Fig. 1:**
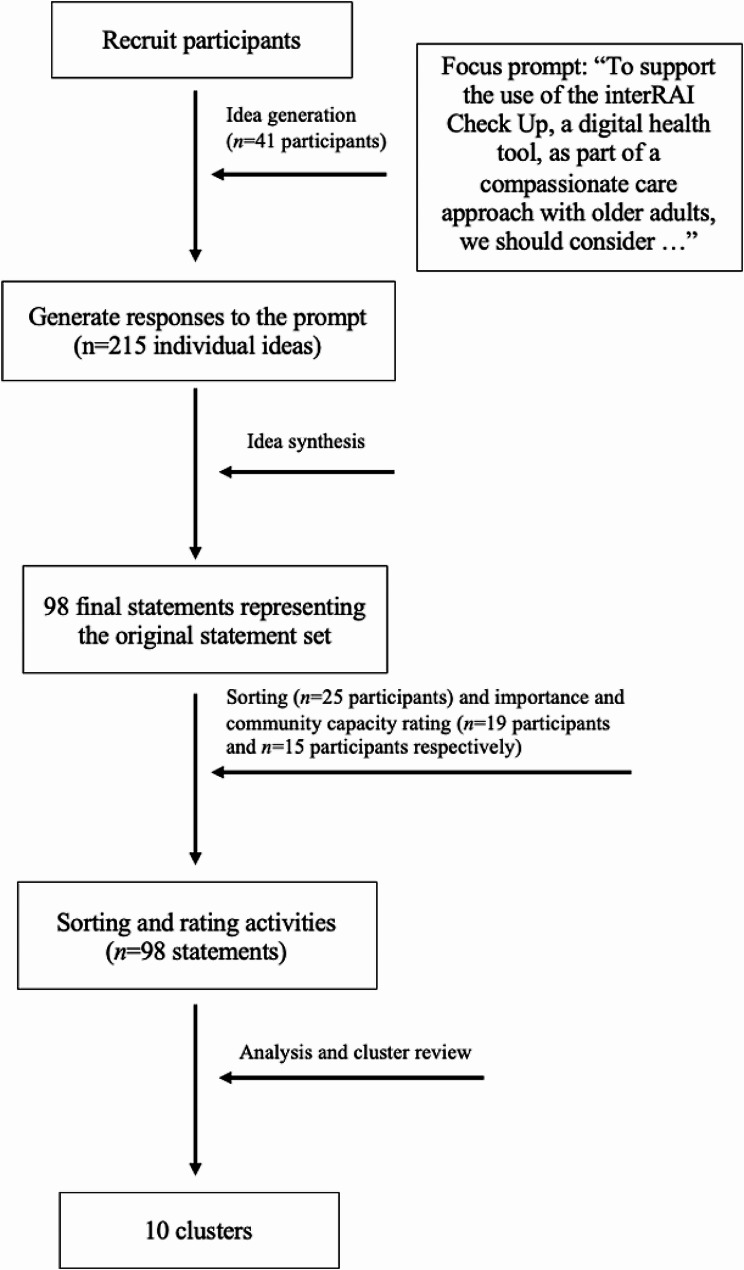
A diagram of the activities involved in group concept mapping and the number of participants who participated in each activity

A total of 41 participants completed the idea generation step (refer to Table 1 for summary of their role or sector). For the sorting and rating session, 25 participants completed the sorting activity, and 19 participants completed the rating activity for importance and 15 for community capacity (refer to Fig. 1 for a schematic of the flow of participation).

**Table 1:** Sector or role of participants (*n* = 41) in the idea generation step

Sector/Role	Number (%)
Older adult/care partner	7 (17)
Home care	6 (15)
Community support services	5 (12)
Specialized geriatric services	6 (15)
Primary care	2 (5)
Digital health/health informatics	2 (5)
Research	1 (2)
Other sector/role	6 (15)
Did not respond	6 (15)

### Cluster map

**Fig. 2:**
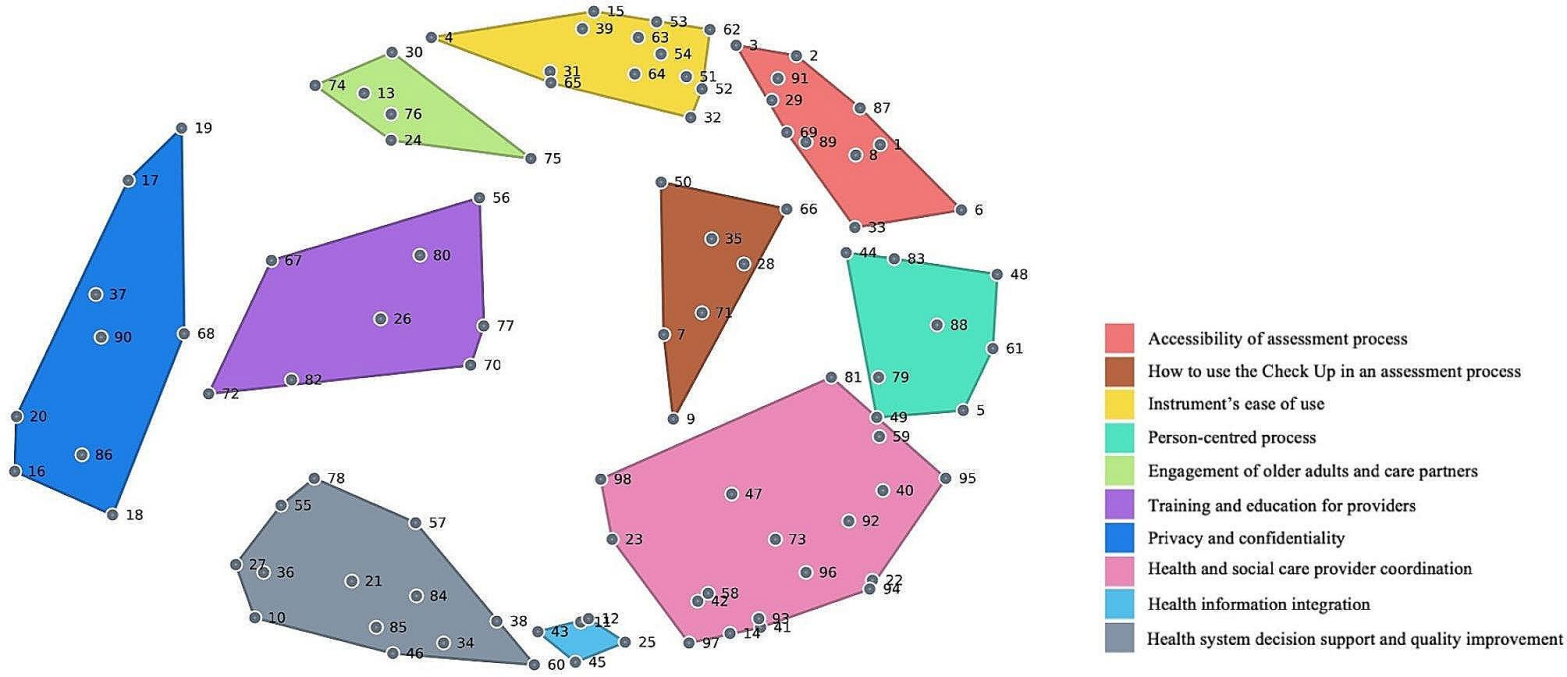
Cluster map represents which statements are contained in each domain. The smaller the cluster, the greater the interrelationship between ideas within the cluster

A ten-cluster map, reflecting all 98 statements, provides a framework of considerations for employing the interRAI Check Up as part of a compassionate care approach with older adults (refer to Fig. 2). The concept map highlights the concepts and the connections between ideas, represented by the distances between the clusters and statements. The smaller clusters indicate that these statements were most consistently sorted together (e.g., *Health Information Integration*). The larger clusters show where participants may have sorted statements together but also with other clusters. For example, statements in *Privacy and Confidentiality* were often sorted with statements from the adjacent cluster, *Training and Education for Providers*. *Health and Social Care Provider Coordination* statements were also sorted with statements in *Person-Centred Process* as provider fragmentation can contribute to care that is experienced as not person-centred. Similarly for the *Instrument’s Ease of Use* and *Accessibility of Assessment Process* clusters statements were sorted together given that for an instrument to be easy to use, it must also be easy to access.

Refer to Table 2 for the cluster names, their associated importance and capacity ratings, and a sample of statements by cluster.

**Table 2:** Cluster map cluster statements and ratings

Cluster Statements Sample	Rating mean (SD)	
	Importance	Capacity
**Engagement of Older Adults and Care Partners** (6 statements)	**3.18 (0.26)**	**2.72 (0.39)**
ensure that older adults and care partners understand the results of the Check Up and how to use the information to support their own wellbeing		
ensure that older adults see value in the tool and its outputs in a way that makes sense to them.		
**Instrument’s Ease-of-Use** (13 statements)	**3.10 (0.37)**	**2.64 (0.29)**
be aware of cultural and language differences to ensure that questions are understood and answered accurately		
ensure Check Up is in an easily read, easily understood format without jargon or acronyms		
make sure language supports/translation are provided where there is a barrier		
offer various types of support for how to use technology (phone or in-person intro, video of how to complete, phone tech support, in-office support) for older adults		
**Accessibility of Assessment Process** (11 statements)	**3.13 (0.38)**	**2.57 (0.44)**
check to see whether the older adult and/or care partner has the capacity to fill out the tool		
ensure that older adults have access to accessibility aids to use a device		
make the interRAI Check Up user-friendly so it does not deter the older adult from completing it as required		
provide options and confirm whether the older adult and care partner would prefer to fill the tool out online, in person, on paper, or over the phone/video		
**Person-Centred Process** (8 statements)	**3.10 (0.25)**	**2.73 (0.31)**
be clear about how the outputs will contribute to or support care (i.e., need identification; triaging of referrals; develop goals for care; identify risk/vulnerability; act an outcome measure)		
ensure the care-planning process is person centered and holistic and identifies what health means to that person.		
**How to Use the Check Up in an Assessment Process** (7 statements)	**2.94 (0.33)**	**2.72 (0.18)**
effectively communicate the process for completing the Check Up (e.g., provider roles and responsibilities, timelines, and older adult roles and responsibilities)		
develop and share resources for both the use of the software and the Check Up with older adults, care partners, and providers		
**Training and Education for Providers** (8 statements)	**3.08 (0.22)**	**2.68 (0.21)**
leverage existing provincial resources to develop and provide education and support to older adults, care partners, and providers on the use of software and the Check Up		
ensure that primary care and other health care providers are knowledgeable / trained on using the tool and are in agreement that it is a useful tool to gather information for quality care		
**Health and Social Care Provider Coordination (18 statements)**	**2.99 (0.35)**	**2.53 (0.39)**
identify referral pathways for older adults based on Check Up outputs and identified needs		
provide access to providers in all sectors to this tool to ensure that every older adult and their care partner are not being asked the same questions		
set a standard time frame by which the most responsible provider must review the Check Up information to follow-up on any high-risk issues identified (e.g., significant mood issue)		
continue to build trust and understanding across the care system and with older adults using the system as this will be essential for partnership and collaboration.		
ensure that primary care and other health care professionals have buy-in that this tool is an important to use as a compliment to care		
ensure the tool does not replace clinical reasoning of very qualified staff		
**Health Information Integration** (5 statements)	**3.19 (0.38)**	**2.45 (0.31)**
sync information between all provider data sources for an older adult (i.e., electronic health records) to avoid duplication of assessment.		
ensure Check Up information and any referrals are made available to primary care provider		
**Health System Decision Support and Quality Improvement** (13 statements)	**2.92 (0.38)**	**2.46 (0.33)**
consider software solutions that seamlessly integrate with various electronic platforms to avoid duplication of assessment		
understand the sustainability cost of funding and access to software for organizations		
consider how the interRAI outcomes would be used into collaborative care-planning (across teams/programs), which includes the person’s goals/priorities as a guiding component.		
ensure the data collected at the community level is used to inform gaps in health care needs of older adults		
**Privacy and Confidentiality** (9 statements)	**3.11 (0.54)**	**2.82 (0.55)**
complete data sharing agreements to support older adults’ privacy, confidentiality and appropriate sharing		
ensure the older adult and their care partner understand who will have access to the information, where it will be stored, and with whom it will be shared		
Ensure the data collected is stored in a secure environment		

Note. Importance and capacity were rated on a scale from 0 to 4. SD = standard deviation

#### Engagement of older adults and care partners

The *Engagement of Older Adults and Care Partners* cluster includes six statements that describe a compassionate care approach to actively involve older adults and their care partners in their health care. The statements focus on the empowerment of older adults in their collaborative relationships with providers by ensuring older adults and their care partners: understand the purpose and results of the Check Up, know how to use the results to support their own wellbeing, and update their own profile and make it accessible to providers.

#### Instrument’s ease of use

The *Ease-of-Use* cluster is one of the largest, comprising 13 statements focused on suggestions to ensure the instrument is easy to use and understandable for older adults and their care partners. The statements focus on the user-friendliness of the software (e.g., easy navigation, simple instructions), provisions for translation into languages other than English, and avoidance of jargon or medical terminology. Providing technological support, as needed, for older adults independently completing the Check Up was suggested in the form of video instructions, phone support, or in-person instruction.

#### Accessibility of assessment process

Closely related to ease-of-use are the 11 statements in this cluster which reflect strategies for enhancing the *Accessibility of the Assessment Process* when using the interRAI Check Up with older adults. Accessibility refers to both access to technology (e.g., devices) and the internet and to supports to participate in the assessment (e.g., hearing aids, voice-to-text recognition), in any setting that the Check Up may be completed (e.g., home, primary care setting, etc.). Other considerations include checking the older adult’s and/or care partner’s capacity to independently complete the Check Up, as well as offering different options for completing the tool, such as online, in-person, on paper, or over the phone/video, and with and without the support of a provider.

#### Person-centred process

There are eight interrelated statements in the *Person-Centred Process* cluster, highlighting the importance of placing older adults’ needs and preferences at the forefront of encounters where the Check-Up would be used. These statements discuss a holistic approach ensuring that the care is tailored to the unique needs of each person. This care approach includes balancing the collection of digital information with face-to-face conversation, maintaining personal interaction as part of the assessment process, ensuring the older adult and their care partner have access to a copy (digital or physical) of the Check Up and its outputs and that they have been provided education on the meaning of those outputs. The focus of this cluster is ensuring that the care-planning process is person- centered and identifies what health means to the individual person even as technology is deployed.

#### How to use the check up in an assessment process

This cluster resides in the middle area of the map, where the clusters are about health- and social-care providers’ use of the Check Up. This cluster consists of seven ideas focused on *How to Use the Check Up in an Assessment Process* with older adults. These statements include considerations for providers when using the Check Up such as, effectively communicating the process for completing the Check Up so older adults understand what to expect and why it is being used. Participants also suggested that providers should determine the older adult population that would be best served by a self-report tool as opposed to a clinician-elicited assessment (i.e., interRAI Home Care) and that resources should be developed for both the use of software and the Check Up for providers and ones to share with older adults.

#### Training and education for providers

Located on the map beside the “how to” cluster is the *Training and Education for Providers* cluster. These nine statements are about equipping health- and social-care providers with the necessary knowledge and skills to effectively integrate the Check Up into their practice. They encompass strategies such as, leveraging existing interRAI provincial resources to develop and provide education on the use of software and the Check Up, providing education on self-management strategies and relevant community support services, and using the outputs of the Check Up in care planning. Establishing a shared understanding of how the Check Up can be used in the care of older adults and their care partners was also suggested as part of the training and education content.

#### Health and social care provider coordination

The three clusters at the bottom of the map capture statements that relate to the system-level use of the Check Up. The *Health and Social Care Provider Coordination* cluster contains the most statements (18) with content outlining how the sharing of the outputs of the Check Up would foster collaboration among health- and social-care providers. Statements include suggestions to develop referral pathways based on Check Up outputs and designate most-responsible providers and timelines to follow-up on any high-risk issues (e.g., significant mood issue). Participants noted that access to the Check Up outputs should be shared with all providers to reduce assessment burden on older adults and duplication of assessment.

#### Health information integration

The five statements in this cluster describe ways to support *Health Information Integration* from various sources into a comprehensive electronic record, allowing providers to have a more complete understanding of an individual’s health. Identified strategies include syncing digital information between all provider data sources, using automated processes (i.e., ‘bots’) to automatically identify older adults with known health issues to streamline care processes, and leveraging existing integration systems that would allow organizations to seamlessly share the interRAI Check Up results within the circle of care.

#### Health system decision support and quality improvement

Thirteen statements were sorted into the *Health System Decision Support and Quality Improvement* cluster and detail strategies to utilize the outputs of the Check Up at a system level to provide decision support as part of continuous quality improvement. Statements with a quality improvement perspective include selecting Check Up outputs for evaluating coordinated approaches to care, considering how this data may support identification of areas where increased service capacity is required, and considering if some of the items in the Check Up could be used as patient-reported outcome measures. Statements also focused on the technological side of quality improvement across the system by considering funding and access to software solutions to deploy and integrate the Check Up to automatically share information across system providers.

#### Privacy and confidentiality

The eight statements in the *Privacy and Confidentiality* cluster centred on the use and sharing of older adults’ health information through the Check Up assessment process. These statements include opportunities to maintain and describe privacy and confidentiality at both the older adult and system level. Participants generated ideas to ensure the older adult understands who has access to their data, where it will be stored, and how it will be used and can make an informed decision about sharing their personal data. To facilitate integrated care, participants noted that data-sharing agreements need to be developed between organizations that account for a broader circle of care than solely health-care providers.

### Cluster ratings for importance and community capacity

The mean importance score for each cluster ranged from 2.92 to 3.19 out of four (refer to Table 2), with the *Health System Decision Support and Quality Improvement* cluster rated as the lowest relative importance and the *Health Information Integration* cluster rated as the highest relative importance. Most of the clusters (*n* = 7) were rated as greater than 3, representing the ‘important’ category on the 4-point Likert scale. With regards to community capacity, the mean scores ranged from 2.45 (*Health Information Integration* cluster) to 2.82 (*Privacy and Confidentiality* cluster). All mean ratings for capacity across clusters were between 2 and 3, reflecting the ‘a little capacity’ and ‘moderate capacity’ ratings. Of note, during and after the rating exercise, a number of participants commented that they found it challenging to rate the community capacity as they felt they did not know enough about the health- and social-care system capacity, for example related to staffing levels or budgetary restrictions.

### Go-zone graph

**Fig. 3:**
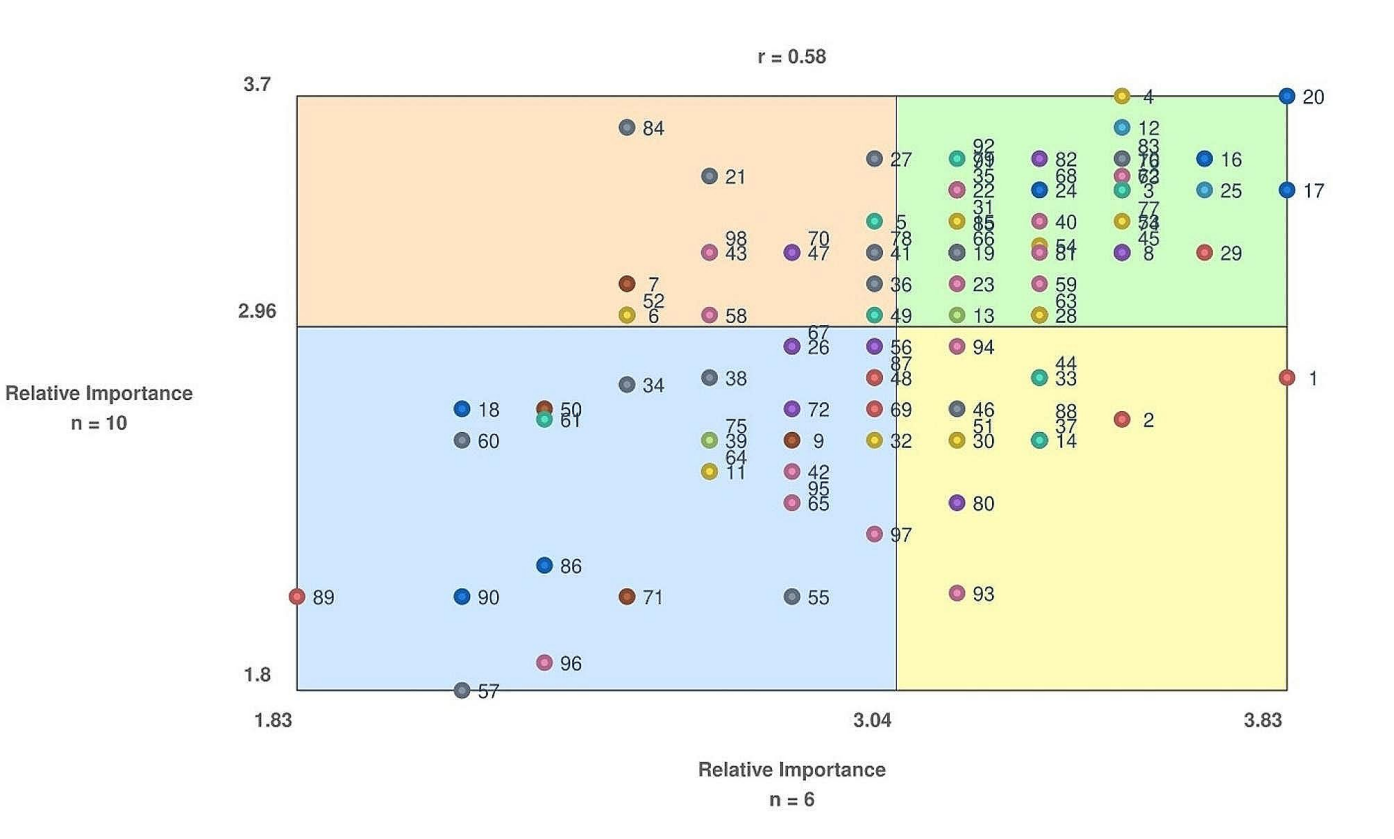
Go-zone graph of priorities comparing older adults (x-axis) to other participants (y-axis). Ratings range from 1 to 4, with 1 indicating low importance, and 4 indicating high importance. Numbers correspond to statement numbers

The go-zone graph displays the agreement between older adults and all other participants related to the importance rating of statements (refer to Fig. 3). Data on the importance rating of all statements was available for only six older adults and 10 other participants. The statements in the left lower quadrant represent agreement on the statements of the lowest relative importance and the statements in the upper right quadrant reflect agreement on the statements of the highest relative importance. The other quadrants contain statements where there was less agreement on importance ratings between older adults and other participants. A total of 35 statements are in the upper right quadrant with representation from all ten clusters and a sample of these statements is reported in Table 2. The rankings by older adults and the other participants were moderately correlated (*r* = 0.58).

Older adult participants rated two statements from the accessibility of the assessment cluster higher compared to all other participants: ensuring that older adults have access to required technology (i.e., devices) and the internet to complete the Check Up. Notably, providers rated items from the decision support cluster regarding shared benchmarking for quality improvement and using the Check Up outputs in care planning, as higher importance than did the older adult participants.

### Action planning strategies

A number of strategies to use the outputs of the interRAI Check Up as part of the integrated care of older adults with multiple chronic conditions were generated based on the set of statements from the go-zone graph that were rated as the highest importance by both older adults and other participants. These strategies are organized by cluster in Table 3. Considerations included strategies for the efficient and integrated administration and use of the Check Up, for example, developing workflow plans for how the Check Up would fit into care processes. Strategies were also developed to foster health- and social-care provider coordination, such as providing education on working as a team based on a shared care plan informed by a standardized self-report instrument.

**Table 3:** *Action plan strategies by cluster*

**Engagement of Older Adults and Care partners**
Determine preferences for completion/debriefing of the Check Up at beginning of assessment with older adults
Determine whom in the older adult’s circle-of-care they would want to receive the Check Up outputs
Develop patient-facing and provider resources for shared decision-making in care planning
Develop patient-facing resources for the older adult to understand how the Check Up outputs relate to suggested health interventions (including discussion of the other tools providers may use)
**Instrument’s Ease-of-Use**
Have translations of the Check Up available in languages common to older adults in the local community
Determine cultural differences related to Check Up items and interpretation of outputs
Work with members of the community to ensure Check Up items and description of outputs are culturally relevant
Have translation services available
Educate providers on translating outputs of assessment into plain language
**Accessibility of Assessment Process**
Determine what accessibility aids are required for the older adult and have accessibility aids available to complete the Check Up and review the outputs
Establish a process for determining if the older adult and/or care partner can successfully complete the Check Up
Before starting the assessment, determine older adult’s preference for completing the tool (independently or with supports and online, over the phone, or in-person)
Select user-friendly software based on: experiences of older adults using the software for the assessment; the presentation of the content and outputs in plain language; ability to complete/access on phones, tablets, computers; ability to complete online and offline; and accessible text size and navigation
**Person-Centred Process**
Identify the older adults’ goals for care planning
Determine as a team how the outputs of the assessment will be used and communicated both to providers and older adults
Determine how the Check Up fits with other clinical screening/assessments and care planning processes across the health- and social-care system
**How to Use the Check Up in an Assessment Process**
Develop workflow (including providers’ roles and responsibilities) for using the outputs of the Check Up
Develop a clear “service charter” related to use of the Check Up (e.g., what clients and care partners can expect)
Develop and share patient-facing resources on using the software and completing the Check Up
Develop education and resources for providers on using the software and completing the Check Up that can be customized at the organizational level
**Training and Education for Providers**
Allow time and opportunities for team development, education and shared decision making.
Develop education modules/sessions on using the Check Up in practice for providers
**Health and Social Care Provider Coordination**
Provide education on working together as a team on a shared care plan informed by a standardized self-report assessment
Provide education on using outputs of Check Up as minimum data set to support comprehensive geriatric assessment
When care planning, determine the most-responsible provider for further assessment and support
Allow time for providers to build relationships when working in new integrated care models
**Health Information Integration**
Consider both health- and social-care sectors in shared software procurement and implementation
Design processes to automatically share information across electronic health records
**Health System Decision Support and Quality Improvement**
Plan for quality improvement at the team and community level (e.g., collect data once, use in multiple ways)
**Privacy and Confidentiality**
Complete data sharing agreements with partner organizations and providers (e.g., Health Information Management Plans)
Develop a standardized process for informed consent related to health- and social-care system data sharing
Define the circle-of-care for integrated health- and social-care delivery by organization/team
Develop education on data-sharing principles of an integrated team

## Discussion

This mixed methods study identified a number of practical strategies related to using the outputs of the interRAI Check Up as part of integrated health and social care for older adults with multiple chronic conditions and their care partners, considering: older adult engagement, instrument’s ease of use and accessibility, assessment process, training and education for providers, care provider coordination, health information integration, health system decision support and quality improvement, and privacy and confidentiality. Participants generated ideas to ensure the maintenance of a compassionate, person-centred approach when leveraging a digital health tool in the care of older adults. Strategies generated from these factors highlight the importance of preparing, intervening, and evaluating at older adult and provider, organizational, and system levels when introducing a new digital health tool. While participants were reflecting on the use of the interRAI Check Up Self Report instrument, the findings have relevance for the use and implementation of other electronic wellness instruments and digital tools.

The group concept mapping activities generated only two clusters about the digital health tool itself (instrument’s ease of use and how to use the Check Up in an assessment process), demonstrating that the instrument’s selection and thoughtful use are important but additional factors beyond the implementation of the instrument need to be considered. This finding is supported by previous research explaining the factors that influence the adoption of technology in health-care settings [35, 36]. To support the integration of new digital tools, Shaw and colleagues [36] suggest that care delivery must be “re-invented”, and they propose a [Tool + Team + Routine] heuristic to guide implementation and sustainment of new digital tools. The team must see value in the ***tool***’s ability to enhance their care of older adults and to make that care more efficient [36]. The ***team*** must also agree that there is an issue in the current care delivery (i.e., fragmented health and social care) and plan for new ways of working together that will incorporate the digital health tool into ***routine*** practice [36]. The *Health- and Social-Care Provider Coordination* cluster, the largest cluster, contained strategies to ensure there is ‘buy-in’ for the use of the Check Up but also intentional planning for coordination amongst providers that may not have historically worked closely together. Careful planning of new practice routines is also important to ensure that introduction of the Check Up into interprofessional team practice does not further fragment care by creating “data silos” [37]. Participants also noted that that it takes time and effort to learn to work as a coordinated, interprofessional team and this finding has been noted in other evaluations of interprofessional teams caring for persons with complex chronic conditions [2, 38]. Strong and trusting relationships in the interprofessional team is regarded as a critical component of workforce capacity in implementing integrated care programs and in the context of these findings, these relationships must extend beyond providers in the health-care sector and with social-care team members [7, 39].

Health information integration is considered a facilitating factor for integrated care and was supported by this study’s findings [7, 13, 14, 17, 40]. The *Health Information Integration* cluster was rated as highly important but with the lowest community capacity rating across the clusters. This cluster also was the smallest (i.e., the fewest number of statements). The lower community capacity and smaller number of statements generated for this cluster may indicate that participants are unsure of how the integration of personal health information could occur across the health- and social-care sectors or reflect the reality that sophisticated data-sharing infrastructures are not in place in their community. Given that rating capacity was noted as challenging for participants, this may be another sign of system fragmentation. Local and other jurisdictions have felt tensions balancing privacy requirements with the benefits of more integrated information-sharing arrangements [41]. In an international survey of integrated care programs, only one-third of programs had secure data infrastructure platforms that supported patient information sharing among providers and many programs relied on team meetings or one-on-one communication to share information [13]. However, this study highlighted that both older adults and care providers rated the cluster of privacy and confidentiality as very important and high community capacity, indicating a community willingness and perceived ability to share information in a secure manner. A recent literature review of patient perspectives on consent related to sharing personal health information digitally noted that patients are willing to share their information to inform their care if it is done carefully, maintaining the privacy and security of their data [37]. Additionally, participants in the included studies wanted clear information on why their personal health information was being collected and shared, with whom it would be shared, and for what purpose [37]. Information technology expertise and support will be critical in moving from traditional forms of data sharing (e.g., team meetings) to more seamless, data infrastructure platforms informed by harmonized information management plans [40, 42–44].

Health information integration is a prerequisite for continuous quality improvement, for example, aggregating information to understand population health needs and health system impact [7, 43]. The *Health System Decision Support and Quality Improvement* cluster was a large cluster in this study, but participants rated the cluster as the relative lowest importance and of low capacity, with older adults rating some of the ideas in this cluster lower than other participants. This may represent another area where providers and older adults are uncertain of the importance of continuous quality improvement to foster a responsive learning health system for the developing needs of the community [11]. In reality, there are relatively few examples in the intersectoral care of older adults with multiple chronic conditions of continuous quality improvement efforts that engage both providers and older adults [16, 45]. The suite of standardized interRAI instruments were designed with this purpose in mind: to not only inform care planning at the person-level but also program planning and cross-sectoral comparisons through embedded aggregate reporting features and quality indicators [46, 47]. The Check Up also has interoperability and shared language with other interRAI instruments, such as those used in home care, given the shared items and outputs, which also facilitates on-going assessments over time and across settings [22]. Implementation planning for the use of the Check Up across health- and social-care sectors will need to include strategies to enact continuous quality improvement at both individual program and system levels.

Participants were able to generate many ideas around using a digital health tool in the care of older adults and their care partners, in contrast to the notion that older adults do not want to use technology in care interactions or that technology interferes with compassionate care [18, 48]. Instead, they proposed ideas to engage older adults in using the Check Up and ensuring an accessible and easy-to-use process, including the provision of the Check Up in an older adult’s preferred language. Older adult participants highlighted the importance of providing access to devices and the internet when using a digital health tool in practice, which is a very relevant consideration given the cost implications for older adults [49]. Recommendations in the *Accessibility* and *Ease-of-Use* clusters are critical to ensuring that use of digital health tools does not create health inequities for older adults, in particular those experiencing socioeconomic disadvantages [49].

### Strengths and limitations

A main strength of this study is its adherence to the group concept mapping methodology, which allows input from persons with diverse perspectives and roles in a local community and the subsequent co-creation of a concept map and action-planning strategies. Group concept mapping has advantages over traditional data collection methods, such as interviews or focus groups, due to its participatory nature and the creation of community-authored visual representations of ideas that guide planning [50]. The study employed a patient-oriented approach with active participation of older adults on the steering committee, increasing the relevance and applicability of the findings. Several limitations should be considered. One of the limitations was the potential for bias in the selection of older adult participants. The researchers recruited these individuals through existing networks (e.g., patient partners on organizational boards), which may not have been representative of the broader population of older adults. The number of older adults (*n* = 6) represented in the go-zone is very small for this type of analysis [28]. Further, some participants struggled with rating the statements on community capacity, which limited the number of responses in this data set. Due to the exploratory nature of this study, future research will be required to validate, refine, and test these strategies in this community and tailor for consideration in other communities. As well, work should be directed to considering how these strategies could be used to inform implementation of electronic wellness instruments and digital tools.

## Conclusion

Older adult and provider, organizational, and system level factors need to be considered when implementing and using the outputs of an electronic wellness instrument across health- and social-care providers. This study extends and supports the existing evidence base on digital health tool adoption by providing a co-created, community action plan from the perspectives of older adults, care partners, health and social care providers and administrators, digital health experts, and researchers. Older adults, care partners, and the care team favourably regarded the use of the Check Up from both self-report and digital perspectives. The breadth of considerations in implementing and sustaining the use of the Check Up, from ensuring a person-centred assessment process to using the outputs as part of continuous quality improvement, would be relevant in the implementation of other electronic wellness instruments and digital tools or the re-implementation of an existing instrument to optimize its potential to support integrated care. This study also showcased a helpful research methodology (GCM) to gain important perspectives in a manner where all voices are heard and valued.

## Data Availability

Data requests should be submitted to the first author.

## Abbreviations

GCM: Group concept mapping
